# Telomere Transcripts Target Telomerase in Human Cancer Cells

**DOI:** 10.3390/genes7080046

**Published:** 2016-08-16

**Authors:** Theresa Kreilmeier, Doris Mejri, Marlene Hauck, Miriam Kleiter, Klaus Holzmann

**Affiliations:** 1 Institute of Cancer Research, Comprehensive Cancer Center, Medical University of Vienna, Borschkegasse 8a, Vienna 1090, Austria; theresa.kreilmeier@gmx.at (T.K.); doris.mejri@meduniwien.ac.at (D.M.); 2 Department for Companion Animals and Horses, University of Veterinary Medicine Vienna, Veterinärplatz 1, Vienna 1210, Austria; miriam.kleiter@vetmeduni.ac.at; 3 Department of Clinical Sciences, College of Veterinary Medicine, North Carolina State University, 1060 William Moore Drive, Raleigh, NC 27607, USA; marlene_hauck@ncsu.edu

**Keywords:** telomerase, enzyme inhibition, telomere, long non-coding transcript, viral expression systems, tumor cell lines, human, canine

## Abstract

Long non-coding transcripts from telomeres, called telomeric repeat-containing RNA (TERRA), were identified as blocking telomerase activity (TA), a telomere maintenance mechanism (TMM), in tumors. We expressed recombinant TERRA transcripts in tumor cell lines with TA and with alternative lengthening of telomeres (ALT) to study effects on TMM and cell growth. Adeno- and lentivirus constructs (AV and LV) were established for transient and stable expression of approximately 130 units of telomere hexanucleotide repeats under control of cytomegalovirus (CMV) and human RNase P RNA H1 (hH1) promoters with and without polyadenylation, respectively. Six human tumor cell lines either using telomerase or ALT were infected and analyzed for TA levels. Pre-infection cells using telomerase had 1%–3% of the TERRA expression levels of ALT cells. AV and LV expression of recombinant TERRA in telomerase positive cells showed a 1.3–2.6 fold increase in TERRA levels, and a decrease in TA of 25%–58%. Dominant-negative or small hairpin RNA (shRNA) viral expression against human telomerase reverse transcriptase (hTERT) results in senescence, not induced by TERRA expression. Population doubling time, cell viability and TL (telomere length) were not impacted by ectopic TERRA expression. Clonal growth was reduced by TERRA expression in TA but not ALT cell lines. ALT cells were not affected by treatments applied. Established cell models and tools may be used to better understand the role of TERRA in the cell, especially for targeting telomerase.

## 1. Introduction

Immortality of tumor cells is a hallmark of cancer and a possible therapeutic target [1]. Most tumors activate telomerase as a telomere maintenance mechanism (TMM) to gain infinite growth capacity [2]. The telomerase reverse transcriptase (*TERT*) gene encodes the catalytic subunit of telomerase. Highly recurrent mutations in the *TERT* promoter were found in over 50 cancer types and are the most common mutations in many cancers, making telomerase activation an attractive target for cancer therapy [3]. 

Transcripts from telomeres termed telomeric repeat-containing RNA (TERRA or TelRNA) were identified first as developmentally regulated RNA originating from RNA polymerase II [4,5]. Telomere transcripts originate in human cells from CpG-island promoters from around half of chromosomal ends [6]. TERRA-mimetic oligonucleotides were identified in cell extracts as inhibitors of telomerase activity (TA) [5]. Furthermore, TERRA transcripts were identified in vitro as natural ligands and direct inhibitors of telomerase [7]. TERRA works mainly via sequestration of telomerase, thus preventing access of the enzyme to the telomere substrate. TERRA is expressed at high levels in non-malignant cells, but at low levels in tumor cells [5,8]. Reduced TERRA expression is associated with TA in tumors and cancer cell lines [8,9]. Moreover, in patients with high-grade astrocytoma and detectable TA, elevated TERRA expression may predict a better prognosis [8]. The relation of TERRA with telomere maintenance and cancer is a vital research topic and described in several reviews [10,11,12,13,14,15]. TERRA may be a promising biomarker and potential tool in anti-cancer therapy [12,13]. Furthermore, such an approach may be independent of species as TA was detected in tumors from other mammals as well [16]. 

We hypothesized that the telomeric part of TERRA may work as a potential telomerase-targeting drug and developed viral systems for transient and stable recombinant expression of telomeric repeats in human cell cultures for further study of this approach, including in additional species such as canine.

## 2. Materials and Methods

### 2.1. Cell Culture

Human tumor cell lines originated from colorectal adenocarcinoma (SW480), from cervical carcinoma (HeLa), from glioblastoma multiforme (T98G, YT-BO), and from osteosarcoma (Saos-2, U2OS). YT-BO was established from tumor tissue at the Medical University of Vienna (Vienna, Austria) and the origin of T98G was recently published [8]. Colon tumor cell lines LT97 and Vaco235 were kindly provided by Brigitte Marian (Medical University Vienna, Austria) and grown as described [17,18]. GM-847 and GM-639 are SV40-immortalized skin fibroblasts, and G-292 osteosarcoma cell lines were kindly provided by Roger Reddel (Children’s Medical Research Institute, Sydney, Australia). All other human tumor cell lines were obtained from the American Type Culture Collection (ATCC, Manassas, VA, USA). Cells were grown at 37 °C under 5% CO_2_ in media with 10% fetal bovine serum (FBS) as recommended: HeLa in Roswell Park Memorial Institute medium (RPMI)-1640, T98G in Minimum Essential Medium Eagle (MEME) medium with 10µl/ml non-essential amino acids (NEAA) and 2 µL/mL pyruvate, Saos-2 and U2OS in McCoy’s. SW480 and YT-BO were grown in RPMI-1640. Canine tumor cell lines originated from soft tissue sarcomas (MBSa, CoFSa, and PSTS), and were obtained from Marlene Hauck and grown as described [19]. 

### 2.2. Adeno- and Lentivirus Constructs

For transient and stable recombinant TERRA expression in adenovirus (AV) and lentivirus (LV) constructs, a 0.8 kbp telomere DNA fragment cloned in pSP73 (Promega, Mannheim, Germany) was kindly provided as pSP73.Sty11 by Yasuhiko Kiyozuka (Kansai Medical University, Osaka, Japan) [20]. The plasmid was established byTitia de Lange (Rockefeller University, New York, NY, USA) [21]. Around 80% of the inserted fragment was successfully validated by sequencing of both strands (VBC Genomics, Vienna, Austria) and contains ≈130 telomeric repeats of both perfect (TTAGGG) and degenerated (TTGGGG) telomere hexanucleotides. Recombinant AV and LV for transient and stable expression of the telomere fragment were constructed using the Gateway cloning system (Invitrogen, Lofer, Austria) with polymerase II (CMV) and III (hH1) promoters and respective termination signals. In brief, pENTR with hH1 promoter and terminator used for small hairpin RNA (shRNA) expression and pAd/CMV and pAd/PL plasmids were kindly provided by Hiroshi Takemori (Osaka University, Japan). Plasmids were ligated by T4 ligase (Fermentas—Thermo Fisher Scientific, Vienna, Austria), transformed in TOP10 Chemically Competent *Escherichia coli* strain (Invitrogen) and grown under kanamycin or ampicillin (Sigma-Aldrich, Vienna, Austria) antibiotic selection pressure. The oligonucleotides for construction of pENTR shRNA were sense 5′-GGCCAGTGGAATTCGAGACCAGCTTCAAGAGAGCTGGTCTCGAATTCCACTTTTTTT-3′ and antisense 5′-AATTAAAAAAAGTGGAATTCGAGACCAGCTCTCTTGAAGCTGGTCTCGAATTCCACT-3′ targeting eukaryotic translation initiation factor 4A3 (*EIF4A3*, alternatively termed *NMP265* [22]) with sequences as published for small interfering RNA (siRNA) [23]. Briefly, single-stranded oligonucleotides were annealed to resulting double-stranded DNA (dsDNA) with sense-loop-antisense structure and respective overhangs for direct cloning into NotI and EcoRI sites of the pENTR-hH1 plasmid. The shRNA structure was removed by restriction with EcoRI (underlined) and after religation to result in pENTR-hH1 with the following recombinant hH1 driven transcript expression cassette: **A**GTGGAATTCCAC**TTTTTTT**. Bases for transcription start and termination are marked in bold. Next, the EcoRI restriction enzyme cloning site (underlined) located between the hH1 promoter and the terminator of pENTR-hH1 was replaced by insertion cloning of the linker oligonucleotide 5′-AATTGTCGAC-3′ with a SalI site (underlined). The inserted telomere fragment was cut out from the pSP73 plasmid by restriction enzymes BamHI and BglII (Fermentas) and inserted in sense and antisense orientation into the novel SalI site of pENTR-hH1 after partial fill in of restriction sites by Klenow fragment (Invitrogen). Single bacteria clones with plasmid constructs of telomere fragment in sense or antisense orientation were identified by restriction analyses of plasmid DNA and comparison with the predicted banding patterns using Clone Manager 9 (Scientific & Educational Software, Cary, NC, USA). From the pENTR-hH1 plasmid with inserted telomere fragment, the H1 promoter was deleted for use with Gateway expression vectors with built-in expression cassettes using CMV promoters, like pAD/CMV and pLenti4/V5 (Invitrogen). To this end, the hH1 promoter was cut out with SacI and BamHI (Fermentas) and the remaining plasmid fragment was treated with Klenow fragment (Invitrogen) to generate blunt ends, ligated and transformed in competent *E. coli* as described before. All pENTR plasmid constructs were validated by sequencing. The inserted fragments of the pENTR plasmids were transferred by Gateway recombination cloning technique (Invitrogen) to adenoviral (pAd/CMV/V5-DEST and pAd/PL-DEST) and lentiviral (pLenti6/BLOCK-iT-DEST and pLenti4/V5-DEST) expression vectors. Recombinant lentivirus was produced by ViraPower Lentiviral Expression System as recommended by manufacturer (Invitrogen). AV expressing shRNA against human telomerase reverse transcriptase (hTERT) was constructed from retroviral vector pMKO-1P-C3 kindly provided by William C. Hahn (Dana-Farber Cancer Institute, Harvard Medical School, Boston, MA, USA) [24]. In brief, an expression cassette for shRNA with U6 promoter targeting region 3114–3134 from the hTERT transcript was cloned into pENTR and transferred into pAd/PL-DEST for AV production as recommended by the manufacturer (Invitrogen). AV expressing dominant-negative hTERT was provided by Silvia Bacchetti (McMaster University, Hamilton, ON, Canada) [25] and AV expressing enhanced green fluorescent protein (eGFP) was used as control [26]. Recombinant AVs were amplified as described [27]. Virus titers were determined by Adeno-X Rapid Titer Kit (Clontech, Saint-Germain-en-Laye, France) and by eGFP fluorescence-activated cell sorting analysis (FACSCalibur, BD Biosciences Clontech, Singapore). Constant numbers of cultured cells were incubated with varying numbers of AV termed multiplicity of infection (MOI) for transient recombinant expression. For stable expression, cell populations were selected after infection with LV Lenti6 and Lenti4 by growing in the presence of lethal doses of antibiotics blasticidin and zeocin (Invitrogen), respectively.

From these cell populations, individual cell lines originating from a single clone were established by serial dilution assays. Cells were counted by CASY (OMNI Life Science, Raynham, MA, USA), diluted and seeded as one cell per well in 96-well microtiter plates. Assays were performed twice to ensure single clonal origin of established cell lines.

### 2.3. Relative Telomere Length and Expression

DNA and RNA were isolated and relative telomere length (TL) as telomeric content and endogenous TERRA expression were determined by quantitative PCR (qPCR) as described [8]. Relative quantity (RQ) values were calculated from two independent experiments. For recombinant TERRA expression the following primers were used: exo-5′_f (5′-CGATCCCCGGGTACCGAG-3′), exo-5′_r (5′-CCCCAACCCCAACCGGAAT-3′), exo-3′_f (5′-TTAGGGTTAGGGTTCGGAAT-3′), exo-3′_r (5′-AAGTGGAATTGTCGATCTGATA-3′), T7mod (5′-TAATACGACTCACTATAGGGAGAC-3′). Reference *36B4* gene primers for human and canine are described [19]. 

Telomeric RNA fluorescence in situ hybridization (FISH) was performed as described [4], except that a peanut agglutinin-fluorescein isothiocyanate (PNA-FITC)-(CCCTAA)_4_ probe (Dako, Glostrup, Denmark) dissolved in a hybridization buffer (70% formamide, 2 μg/μL bovine serum albumin (BSA), 10% dextran sulphate in 2× saline sodium citrate) was used for visualization of TERRA (UUAGGG)-repeats. Slides were dehydrated and mounted in Vectashield mounting medium (Vector Laboratories, Burlingame, CA, USA) with 0.5 μg/mL 4′,6-diamidino-2-phenylindole (DAPI) for immunofluorescence microscopy as described previously [28].

### 2.4. Telomerase Activity

Protein extracts for TA analyses were prepared from cells as described [8]. TA was quantified as total product generated (TPG) units by qPCR-telomeric repeat amplification protocol (TRAP) [29] with minor modifications. In brief, reactions were setup ice-cooled in 8-μL volume with GoTaq PCR Master Mix (Promega) including 0.6 μg protein extracts. TA was detected with 200 nM of telomerase substrate (TS) and anchored return CX (ACX) primers. RQ-TRAP was performed on ABI PRISM 7500 Fast Sequence Detection System (Applied Biosystems, Foster City, CA, USA) under the following conditions: incubation for 30 min at 30 °C, followed by a two-step qPCR with 42 cycles of 30 s at 95 °C and 30 s at 60 °C. Relative TA values were converted into TPG units using a standard curve generated by dilution series of telomerase substrate oligonucleotide TSR8 with eight telomeric repeats. Primers and oligonucleotides are described in [8]. One TPG unit corresponds to 0.001 amoles of TSR8 extended for 30 min at 30 °C. Furthermore, qPCR-TRAP results were validated by polyacrylamide gel electrophoresis (PAGE)-TRAP [8].

### 2.5. Cell Growth and Viability

Population doubling level (PDL) and time were calculated based on constant cell numbers seeded with 2 × 10^5^ cells in 6-well plates and counted after seven days by CASY (Omni Life Science) as described [30]. Cell viability rates were determined as recommended using the colorimetric 3-(4,5-dimethylthiazol-2-yl)-2,5-diphenyltetrazolium bromide (MTT) assay (Easy4U, Biomedica, Vienna, Austria) as described [19]. In brief, aliquots of 2 × 10^3^ cells were seeded into 96-well plates in 100 μL cell line specific growth medium. Cells were screened under the phase-contrast Nikon inverted microscope Eclipse TE300 with a TE-FM Epi-Fluorescence attachment (Nikon, Vienna, Austria) 96 h after seeding and the MTT assay was performed in quadruplicates. Colorimetric data were used to calculate cell viability rates. Experiments were repeated twice.

### 2.6. Clonogenicity Assay

Cells were seeded in 6-well plates with 3 mL cell line specific growth medium at a density of 100 cells per well. Unattached cells were removed 24 h later and the cultures were then left to grow for seven days. The number of macroscopic visible colonies was assessed after staining with crystal violet as described [31]. Experiments were repeated for a total of three times.

### 2.7. Senescence-Associated β-Galactosidase Activity

Similar as for PDL, 2 × 10^5^ cells were seeded in 6-well plates with 3 mL cell line specific growth medium, but treated with AV constructs before the senescence-associated β-galactosidase assay was performed as described [32]. In brief, cells were washed with phosphate-buffered saline (PBS), fixed with formaldehyde/glutaraldehyde buffer, washed and incubated at 37 °C with acidic pH buffered staining solution for 4 to 6 h. Blue color development was assessed by phase-contrast microscopy. Representative sections with stained and non-stained cells were counted and percentage of blue stained cells was calculated.

### 2.8. Statistical Analysis

Computations were performed with GraphPad Prism version 5 software (San Diego, CA, USA). Mann-Whitney U test was used for RQ group comparisons. A *p* value ≤ 0.05 was considered significant.

## 3. Results

### 3.1. TERRA Expression in Human Cell Models

**Figure 1:**
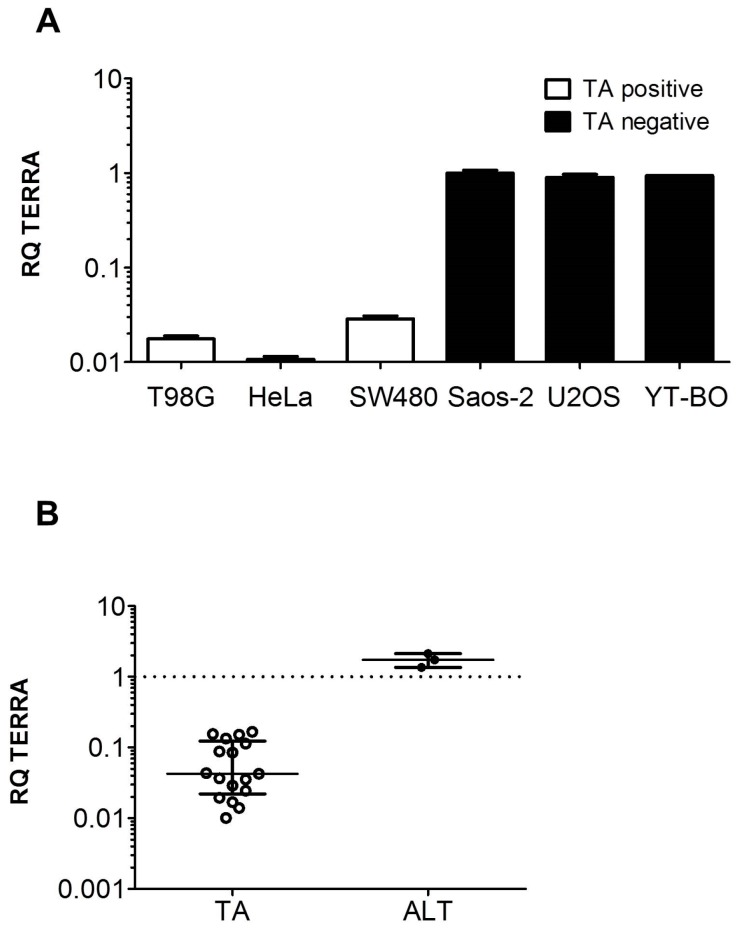
Telomeric repeat-containing RNA (TERRA) transcript expression levels in tumor cell models. (**A**) Bars depict mean and error bars represent standard deviation (SD) of TERRA relative quantity (RQ) values as determined by quantitative PCR (qPCR). RQs are normalized to *36B4* as reference gene and Saos-2; (**B**) Scatter blot of cell lines positive for telomerase activity (TA) (*n* = 6 from colon tumors: SW620, LT97, HT29, HCT116, Vaco, Caco; *n* = 11 from brain tumors: CRL-1718, CRL-2020, KG-MH, KM-YH, LN-140, MGC, MR1, U-373MG, YU-PM, HTB138, HTB-186) and alternative lengthening of telomeres (ALT) (GM-639, GM-847, G-292). RQ value of Saos-2 is indicated by dashed line. Lines depict median and interquartile range.

Tumor cell lines with TA or ALT were analyzed for TERRA expression by qPCR (Figure 1). All tumor cells with TA showed less TERRA expression compared to tumor cells without TA. In detail, TERRA RQ levels of the TA positive cell lines T98G, HeLa and SW480 were 1.8%, 1.0%, and 2.9%, respectively, as compared to TA negative cell line Saos-2 (Figure 1A). These results were further supported by the study of additional TA positive (*n* = 17) and negative (*n* = 3) cell line models (Figure 1B). TERRA levels in TA positive cell lines as compared to Saos-2 were between 1.0% and 17%.

### 3.2. Transient and Stable Expression of Recombinant TERRA in Human Cell Lines

**Figure 2:**
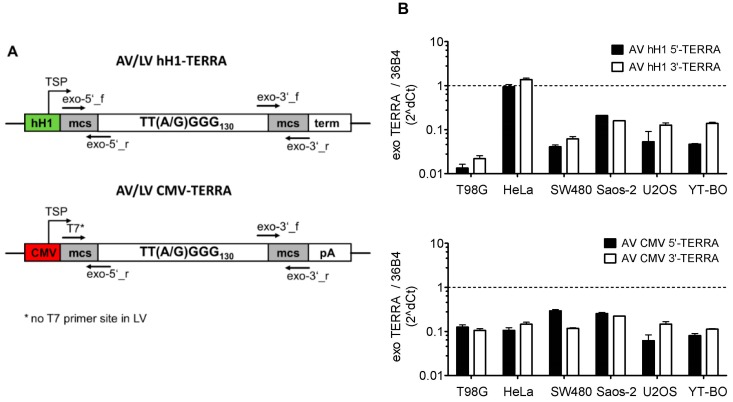
Recombinant TERRA expression constructs in human cancer cells. (**A**) Expression cassettes of adeno- and lentivirus constructs (AV and LV) with specific exo-5′ and exo-3′ primer pairs indicated for detection of recombinant TERRA by qPCR. TERRA expression constructs contain ≈130 telomere hexanucleotide repeats of TTAGGG and TTGGGG variant sequences under control of human RNase P RNA H1 RNA polymerase III promoter (hH1) or human cytomegalovirus RNA polymerase II promoter (CMV). Transcription start point (TSP) and orientation is indicated at hH1 and CMV promoters marked by green and red boxes, respectively. Polymerase III termination site (term), SV40 polyadenylation signal (pA) and multi-cloning-site (mcs) are indicated; (**B**) Recombinant 5′- and 3′-TERRA levels in tumor cell lines 48 h after AV infection with multiplicity of infection (MOI) 10. Recombinant TERRA expression levels from hH1 (AV hH1-TERRA, upper panel) and CMV (CMV-TERRA, lower panel) promoter construct were determined by qPCR in relation to 36B4. Bars depict mean and error bars depict SD.

We infected human tumor cell lines with recombinant AV and LV constructs for expression of ectopic TERRA transcripts with and without polyadenylation (Figure 2). Primers from the 5′ and 3′ multicloning site regions specific for exogenous TERRA were used to quantify recombinant TERRA by qPCR (Figure 2A). After infection with recombinant AV constructs, the relative quantity values of exogenous TERRA transcript levels were determined in relation to *36B4* as reference gene. Transcript levels of TERRA expressed from CMV and hH1 promoters were highly abundant and similar to transcript levels of the ribosomal reference gene (Figure 2B). Results of low variability between 5′- and 3′-TERRA levels indicate that recombinant TERRA was expressed as full transcript without termination. Furthermore, reverse transcription reactions using random hexanucleotide and oligomer dT primers on RNA resulted in similar relative quantities by qPCR of TERRA expressed from CMV constructs (Doris Mejri and Klaus Holzmann, unpublished work). 

**Figure 3:**
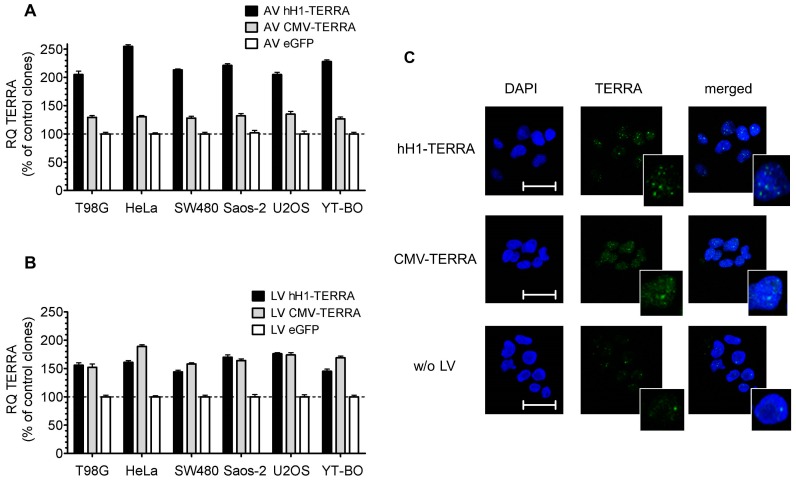
Increase of TERRA levels by recombinant expression. CMV and hH1 TERRA promoter constructs for expression of polyadenylated and non-polyadenylated transcripts, respectively. (**A**,**B**) RQ values for TERRA expression were analyzed by qPCR and compared to *36B4* as reference gene and to enhanced green fluorescent protein (eGFP) infected cells as control; (**A**) Cells were infected with AV at MOI 10 and analyzed after 48 h; (**B**) Cells were infected with LV and single cell clones were isolated. Representative results in one of three clones per cell line at passage four are shown; (**C**) TERRA expression detected by telomeric RNA- fluorescence in situ hybridization (FISH) with fluorescein isothiocyanate (FITC) label in interphase nucleus (4′,6-diamidino-2-phenylindole, DAPI) of LV clones from SW480 cells at passage four. Cells were treated with 200 U/mL DNase A. Scale bars of fluorescence micrographs represent 10 μm. Inlays depict a representative nucleus.

Transient expression of recombinant TERRA from AV constructs did not affect cell viability in the human tumor cell lines studied. Next, cell clones were established by drug selection and two rounds of serial dilution purification from LV infected cell lines. We analyzed TERRA expression of AV infected cells and of selected cell clones after LV infection (Figure 3). All tumor cell lines with expression of recombinant TERRA also showed increased TERRA levels. Short-time ectopic TERRA expression from AV with the CMV promoter resulted in a moderate TERRA transcript increase of 27%–35%, in contrast to a strong 105%–155% increase if ectopic TERRA was expressed from the hH1 promoter (Figure 3A). Cell clones with long-time stable expression of ectopic TERRA from LV increased TERRA levels by 44%–89% independent of the promoter system used (Figure 3B). LV cell clones with recombinant TERRA expression showed a moderate increase of signal in the nucleus but not the cytoplasm by RNA in situ hybridization (Figure 3C). In detail, both ectopic TERRA transcripts expressed by CMV or hH1 promoters with or without poly(A) tail increased the number of TERRA foci compared to control in SW480 cells. Additionally, ectopic TERRA transcription with poly(A) tail compared to transcription without poly(A) tail showed higher staining intensity of the nucleoplasm. 

### 3.3. Telomere Length and Cell Growth Behavior

**Figure 4:**
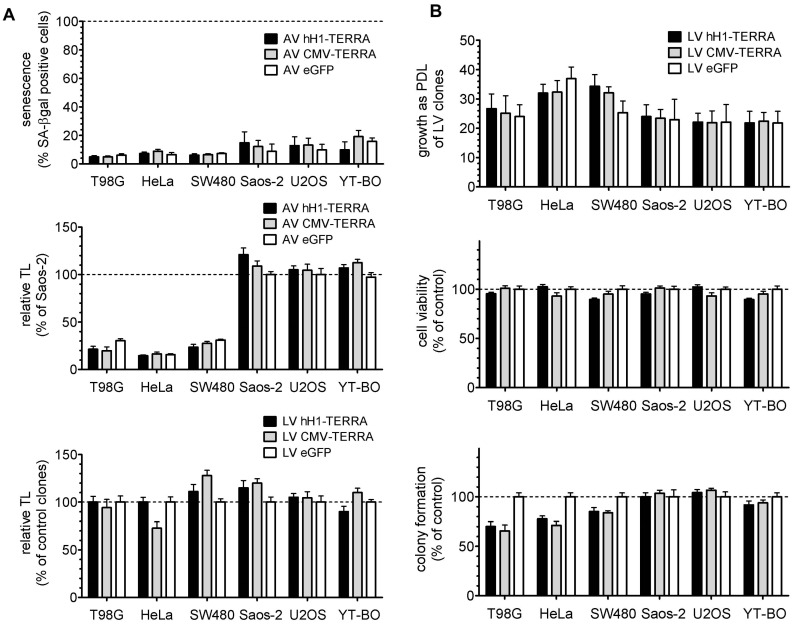
Telomeres and in vitro cell growth properties of tumor cells with recombinant TERRA expression. Bars depict mean and error bars depict SD of two independent experiments performed in triplicates. (**A**) Senescence-associated (SA) β-galactosidase (βgal) positive cells (upper panel) and relative TL analyzed for telomeric content by qPCR as telomere-specific/single-copy gene (T/S) quantity in cells infected with AV at MOI 10 after 48 h (middle panel). Relative TL was analyzed in single LV cell clones at passage four (lower panel). Results for TL were normalized on Saos-2 infected with eGFP (middle panel) and on LV cell clones expressing eGFP (lower panel); (**B**) LV cell clones at passage four were simultaneously grown for six passages in culture and cumulative population doubling levels (PDLs) are shown (upper panel). Cell viability and colony formation values were normalized on LV cell clones expressing eGFP marked by dashed lines (middle and lower panel).

We studied the effects of recombinant TERRA expression on telomeres and on in vitro tumor cell properties (Figure 4). Infection of tumor cell lines with AV constructs for transient expression of recombinant TERRA did not trigger cellular senescence in the human tumor cell lines studied. Recombinant TERRA expression by AV and LV constructs showed effects ±25% on relative TL in individual cell line models (Figure 4A). However, expression of ectopic TERRA resulted in no general trend on the telomeric content. LV clones at passage four after serial dilution purification were analyzed for cell growth, cell viability and clonogenicity as an ability to form in vitro multicellular colonies (Figure 4B). Cell growth capacity as cumulative population doubling level and cell viability by MTT assay were not changed by ectopic TERRA expression in all the tumor cell lines studied. In contrast, clonogenicity was reduced by TERRA expression to 66%–85% exclusively in the tumor cell models with TA. Thus ectopic TERRA expressed with and without polyadenylation inhibits clonal growth capacity of tumor cells dependent on TA.

### 3.4. Telomerase Activity in Cell Models with Recombinant TERRA

**Figure 5:**
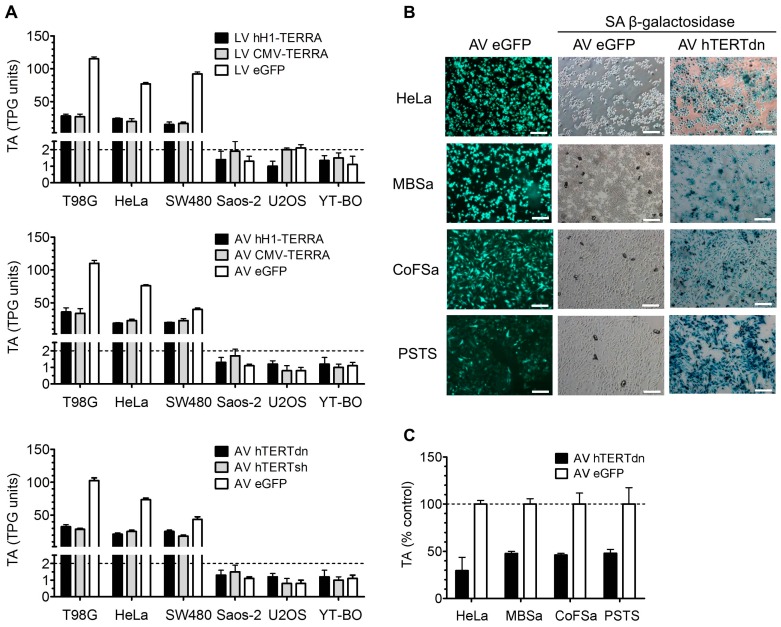
TA in tumor cell lines with recombinant TERRA expression and with TERT-blocking AVs. Bars depict mean and error bars depict SD of two independent experiments performed in triplicates. (**A**) LV cell clones at passage four (upper panel) and cell lines infected with AV at MOI 10 after 72 h (middle and lower panels) analyzed for TA in total product generated (TPG) units by telomeric repeat amplification protocol (TRAP) assay. Dashed lines represent the detection limit; (**B**) Human (HeLa) and canine (MBSa, CoFSa, PSTS) tumor cells infected with AV at MOI 10 were analyzed after 72 h for eGFP expression (green) by fluorescence microscopy and for SA β-galactosidase activity (blue) by phase-contrast microscopy. Scale bars depict 500 μm; (**C**) Human and canine tumor cells analyzed for TA by TRAP assay and normalized to AV eGFP as control.

Both recombinant TERRAs with and without polyadenylation expressed by CMV and by hH1 promoter resulted in strong inhibition of TA (Figure 5). Ectopic TERRA by LV and AV constructs decreased telomerase activity in all TA tumor cell models to 15–27 and 19–36 TPG units, respectively (Figure 5A). Tumor cell models without TA remained TA inactive. Transient TERRA expression from AV constructs in T98G, HeLa and SW480 cells reduced TA after 72 h compared to controls to 31%–33%, 25%–30% and 50%–58%, respectively. This finding is very similar to a decrease of TA as observed with dominant-negative or shRNA AV constructs against hTERT. However, the TA inhibitory mechanism by TERRA is different, as transient knock-down of hTERT transcripts or blockage of the telomerase holoenzyme by expression of mutated hTERT causes senescence in TA addicted tumor cell lines (Figure 5B). The established TERRA cell models can be used to decipher the function of TERRA in human tumor cell lines as well as those from other species. Expression of mutated hTERT in canine sarcoma cell lines with TA resulted in senescence and in inhibition of TA, similar as observed in human cervical carcinoma cells (Figure 5B,C). Screening of a small panel of different canine tumor cell lines (*n* = 8) with TA demonstrated less than 1% of TERRA expression levels compared to human tumor cells without TA (Theresa Kreilmeier, Miriam Kleiter and Klaus Holzmann, unpublished work). TERRA levels in canine tumor cells were comparable to levels in human cells with TA (Figure 1). Whether ectopic TERRA transcripts expressed in canine cells work like in human cells, tumor cells remain to be investigated. These preliminary results suggest the application of canine TERRA cell models for comparative research between human and canine tumor and cell lines.

## 4. Discussion

We developed recombinant viral tools for transient and stable recombinant expression of telomeric repeats in human cell culture to target telomerase activity. Application to telomerase positive tumor cell lines from various origins like glioblastoma and cervical and colorectal carcinoma showed that ectopic expression of the telomeric part from the long non-coding RNA TERRA partially blocks telomerase activity. 

Telomeres and telomerase play a distinct role in aging and cancer, making them very attractive for novel cancer therapies [33]. Furthermore, telomerase was recognized recently to be a central regulator of all of the hallmarks of cancer and thus becomes a strong strategic focus as a therapeutic target in human cancer [34]. There are few telomerase-directed therapies, but telomerase targeting in cancer remains a challenge as anti-telomerase therapies remain unproven. Effectiveness of such drugs on tumor cell growth theoretically depends on the initial length of telomeres until the telomeres shorten to the critical length which initiates growth arrest or death. Such drugs will most likely prove useful for maintenance therapy, rather than a first-line therapy, to control the microscopic residual disease. Phase II clinical trials report toxicities due to effects on some hematopoietic proliferative cells that exhibit regulated telomerase activity [35]. These off-target effects have led to the repurposing of these drugs to treat patients with essential thrombocythemia [36] and myelofibrosis [37]. These studies revealed that current drugs may be non-specific in their effect as no changes in telomere lengths during treatment were observed and the initial telomere lengths did not predict clinical response. New approaches are based on small-molecule telomerase substrates that induce telomere uncapping and promise fewer side effects, as this strategy may result in telomere dysfunction independent of the initial telomere length [38]. Our data indicate that TERRA directly targets telomerase in tumor cells without any further in vitro effects except decreased clonogenic growth. The mechanism of TERRA action is thus clearly different to other telomerase and tumor cell targeting approaches. The established tumor cell lines with modulated TERRA expression are not affected in growth capacity and might thus serve as a model for screening of drugs in combination with telomerase inhibition. Furthermore, possible negative consequences of TERRA upregulation, such as genomic instability and toxicity in the hematologic system, can be evaluated. 

In our study, we successfully validated telomerase inhibition by expression of the recombinant telomere part of TERRA in tumor cell lines. Inhibition was independent if ectopic TERRA was expressed with or without polyadenylation signals, representing the two known isoforms [4]. Endogenous TERRA is largely transcribed by RNA polymerase II [5]. About 7% of TERRA transcripts are 3′ end polyadenylated like most RNA polymerase II transcripts, with some indications that other polymerases like RNA polymerase I and III also transcribe TERRA [4,5]. The 5′ end of TERRA contains 7-methylguanosine cap structures which, together with the poly(A) tail, contribute to its stability. Here, we identified that recombinant ≈800 nt TERRA, expressed by CMV promoter specific for RNA polymerase II and polyadenylated by SV40 signals or by hH1 promoter for RNA polymerase III without polyadenylation, both inhibited telomerase activity.

Recombinant TERRA localize within the nucleus similar as described for endogenous TERRA [4]. Furthermore, different numbers of telomere-associated TERRA foci were detected in ALT and TA cells with high and low TERRA levels, respectively. In detail, 80% to 100% of U2OS cells using ALT as TMM displayed 20 to 40 foci compared to 3 to 7 foci in approximately 30% of HeLa cells using TA. We observed in SW480 cells with TA and low endogenous TERRA levels that ectopic TERRA expression increased the TERRA levels and also the number of TERRA foci. Similarly, increase of TERRA foci was reported if endogenous TERRA became displaced from telomeric chromatin [4]. Whether recombinant TERRA transcripts overexpressed in cells with TA become associated with telomeric chromatin seems to depend upon polyadenylation. Ectopic expression of TERRA with polyadenylation in SW480 cells increased the non-telomere-associated TERRA in the nucleoplasm. Our results correspond with the observation that only unpolyadenylated TERRA is associated with telomeric chromatin [39].

Moderate 1.3–2.6-fold increase of the TERRA levels by AV and LV tools resulted in telomerase inhibition in the target cell lines. This increase by recombinant TERRA expression is remarkably weak, as tumor cells with TA compared with ALT demonstrated significantly lower TERRA expression levels of only a few percent. Our results of higher TERRA levels in ALT compared to TA cell lines are in line with published data of mammalian and yeast cells [6,9,40,41,42,43]. Direct targeting of telomerase in human and canine tumor cell lines by dominant-negative or shRNA AV against hTERT showed very similar partial telomerase inhibition rates as ectopic TERRA expression and suggests a related mechanism of action, but this remains to be proven by further studies. Similar inhibition of canine telomerase enzyme by dominant-negative hTERT is not surprising as the canine TERT protein has one of the highest levels of sequence homology to human TERT among mammals [44]. Furthermore, preliminary experiments with canine TA tumor cell lines demonstrated similarly low TERRA levels. Dogs have been recognized as a model for telomerase and telomeres in cancer research [16,45,46]. As canine telomere biology more closely resembles that of human than, for example, the widely-used mouse, the domesticated dog could provide a useful model system for TERRA studies in the future.

Indeed, TERRA-mimetic oligonucleotides and transcripts were identified as competitive and uncompetitive inhibitors for telomeric DNA by directly binding to telomerase to inhibit telomerase activity in *cis* [5,7]. Furthermore, the ectopically expressed telomeric parts of TERRA might function as decoys to block localization of telomerase enzyme to short telomere ends as recently suggested [47,48]. TERRA might also alter expression levels of essential genes important for telomerase activity, like hTERT and telomerase RNA component (TERC or hTR). Dominant-negative or knock-down of hTERT in some tumor cells results in a decrease of telomerase activity and inhibition of cell proliferation and apoptosis [49,50,51], but failed to block telomerase activity in others [52,53]. We observed large numbers of senescent cells within the studied cell lines shortly after expression of dominant-negative or shRNA AV against hTERT, but this was not observed with ectopic TERRA expression, indicating a very different method of telomerase inhibition or inhibition of functions by TERRA beyond telomerase. 

Population doubling time, cell viability, and telomere length were not affected by ectopic TERRA expression in the tumor cell lines studied. Individual LV cell clones were grown for up to 30 PDL without any indication of a change in cell viability and telomere length. However, clonogenic growth capacity was reduced in TERRA-expressing LV clones from TA but not from ALT cell lines. Telomeric DNA and TERRAs self-associate in vitro to form four-stranded structures called G-quadruplexes (G4) which are non-canonical nucleic acid secondary structures [54,55]. Expression of G4-forming RNA was recently shown to influence cellular behavior through the regulation of gene expression [56]. G4-forming sequences including TERRA may regulate the expression of many genes through the formation of chromatin loop structures via telomere position effect over long distances [57].

The established tools and cell models might be valuable for pre-clinical proof of principle validations targeting telomerase, especially in combination with other therapeutic approaches. Furthermore, the tools and cell models may be applicable to other species such as canine for comparative research.
